# The Associations of Active Travel to School With Physical Activity and Screen Time Among Adolescents: Do Individual and Parental Characteristics Matter?

**DOI:** 10.3389/fpubh.2021.719742

**Published:** 2021-12-15

**Authors:** Caihong Huang, Aamir Raoof Memon, Jin Yan, Youliang Lin, Si-Tong Chen

**Affiliations:** ^1^School of Physical Education, Hubei University of Technology, Wuhan, China; ^2^Institute of Physiotherapy and Rehabilitation Sciences, Peoples University of Medical and Health Sciences for Women, Nawabshah, Pakistan; ^3^Priority Research Centre in Physical Activity and Nutrition, School of Education, University of Newcastle, Callaghan, NSW, Australia; ^4^Department of Physical Education, Wuhan University of Technology, Wuhan, China; ^5^Institute for Health and Sport, Victoria University, Melbourne, VIC, Australia

**Keywords:** active travel, age, geographical location, parents, physical activity, school-aged children, screen time, sex

## Abstract

Little is known about the relationship of active travel to school (ATS) with physical activity (PA) and screen time (ST) by individual and parental characteristics among adolescents, especially in China. To address the research gap, this study aimed to explore the difference of sex, age, living environment, parental occupation and education level in the relationship of ATS with PA and ST among students of grades 7–12 (aged 10–18 years) using cross-sectional data. In 13 cities of Hubei province, China, students from 39 public schools were recruited to engage in the survey. In total, 5,898 students (response rate = 89.6%) were invited into this study. Participants were required to report their ATS (including its types), PA and ST as well as sociodemographic information using a validated questionnaire. Descriptive analyses were used to report the information of all variables. Regression models were used to analyse the relationships of ATS and its types with PA and ST. In a total of 4,128 participants (boys: 50.9%; younger adolescents: 61.9%) included in the final analysis, the proportion of those with ATS was 47.3%. Regarding the types of ATS, walking accounted for over 30%, while cycling was 13.2%. Participants with ATS were more likely to have sufficient PA (OR = 1.26, 95% CI: 1.14–1.39), especially among boys, younger adolescents and those with lower parental education level. However, ATS was not associated with ST (OR = 0.94, 95% CI: 0.86–1.01). Participants with cycling had a higher odds ratio of being physically active (OR for cycling = 1.47, 95% CI: 1.27–1.70; OR for walking = 1.18, 95% CI: 1.06–1.32). The association of ATS types with PA and ST differed by gender, age, living environment and parental educational level as well as occupations. ATS may be a useful approach to increase PA among adolescents, but this should be explained by individual and parental characteristics.

## Introduction

A markedly ubiquitous international trend is that only a small proportion of adolescents fulfil the pervasively recognised physical activity (PA) and screen time (ST) guidelines (1–4). Recent data based on objective measures indicate that just 44.1% of children and adolescents met the PA guidelines [≥60 min of moderate to vigorous PA (MVPA)], while 39.3% of participants met the ST guidelines (<2 h per day) (5, 6). Conversely, meeting the PA and ST recommendations is correlated with more desired health outcomes (7–10). For example, higher PA levels were conversely related to (and greater time spent in ST was positively related to) the odds ratio of being obese (11). For more desirable health outcomes, concurrently promoting PA and discouraging ST among adolescents is an essential public health action.

Given the insufficient PA and higher ST among adolescents globally, researchers have sought effective interventions to change these harmful health behaviours (12, 13). To more effectively promote PA and limit ST among adolescents, active travel to school (ATS) plays a significant role. ATS is an essential source of PA behavioural components among adolescents. Well-documented evidence has presented how ATS is positively correlated with PA among adolescents (14). For example, Roth et al. (15) demonstrated that children and adolescents participating in walking or cycling (types of ATS) were more likely to meet the PA recommendation (OR = 1.31). A further recent investigation of Ecuadorian students similarly demonstrated that participants engaged in ATS were ~3 times more likely to be engaged in sufficient PA as opposed to their peers (16, 17). Comparable results were established in another study conducted in the United Kingdom (17). Overall, the extant research supports the position that ATS can promote PA among adolescents. However, concerning the relationship of ATS with ST among adolescents, an ambiguous relationship remains (14). For example, certain researchers have posited that active travellers among adolescents had a reduced chance of spending time in ST (18–20), although this conclusion contrasted with other studies (21, 22). Collectively, the relationship of ATS with ST should be clarified further, drawing on the accumulated evidence. By drawing on the available literature, ATS may pose an effective means of increasing PA and decreasing ST, albeit with the relationship between ATS and ST being ambiguous (14).

With the increasing importance of ATS on overall PA, Chinese researchers have been devoted to ATS-related studies among Chinese young people. For example, Sun et al. (23) explored the health benefits of ATS, including lower body mass index and lower odds of depressive symptoms among nationally representative Chinese samples. In a cross-sectional study, Yang et al. (24) reported that there was a decline in ATS among 6- to 17-year-old Chinese students. Also, some recent studies identified the social or environmental factors of ATS, such as gated communities, intersection density, the presence of pedestrian streets and so on (25, 26). Within the limited literature, few studies investigated the relationship of ATS with PA and Gao et al. (27) reported that using a pedometer device, male schoolchildren with more steps during before- and after-school time showed higher levels of activity time, while female schoolchildren who had more steps during after-school time reported high activity. In Hong Kong, Huang et al. (28) found that ATS was related to maintaining a relatively higher level of PA. However, little is known about the relationship of ATS with ST among Chinese young people. Despite studies having presented evidence regarding the relationship of ATS with PA and ST, several research gaps still exist. One gap concerns the fact that the recent studies concerning ATS and PA, as well as ST, were based on adolescents from western countries (14, 23), whereas there is limited understanding about them concerning Chinese adolescents. Since evidence suggests that Chinese adolescents are insufficiently active (24), it is anticipated that ATS offers the potential to enhance their PA based on the findings of western studies. Such a relationship has not been investigated conclusively among Chinese samples. Understanding the relationship of ATS with PA is conducive to designing efficient interventions for enhanced PA among Chinese adolescents. Additionally, the correlation between ATS with ST continues to be unclear; thus, this correlation is worth investigating through additional studies (14). In this regard, replicating comparable studies is necessary to advance our knowledge of PA behaviour, which may also provide strategies for reducing ST among Chinese adolescents.

The ATS, PA, and ST are associated with substantial differences in individual and parental characteristics among adolescents. For example, Yang et al. (24) reported that adolescent ATS was significantly higher among younger groups, participants with lower educational attainment, or those residing in rural areas. Certain studies in western countries established that socioeconomic status parameters, for example living environment, parental education, and working status, were linked with ATS among adolescents (15, 17, 19). Such variables are associated with PA and ST among adolescents following the Social Ecological Model (29). Given the correlations between ATS, PA, ST, and socio-demographic variables, the correlation of ATS with PA and ST among adolescents may vary according to their individual and parental characteristics. As suggested in the review by Lubans et al. (30), investigating the overall relationship of ATS with outcomes (e.g., fitness or lifestyles behaviour) ignores the wider personal or social level correlates of behaviour. Therefore, it is necessary to investigate the correlation of ATS with PA and ST further, following the individual and parental characteristics. Nevertheless, across the limited literature, less research has scrutinised the relationship of ATS with PA and ST about socio-demographic factors. For instance, one study indicated that the relationship of ATS with PA was only significant among younger adolescents in Ecuador (16). Another study found that sex is a potential moderator of the relationship between ATS and PA among children and adolescents (21). Unfortunately, there is a dearth of the relevant evidence in this regard from China, which restricts our comprehension of the relationship of ATS with PA and ST among Chinese adolescents. Furthermore, there is limited understanding concerning investigations into the role of parental characteristics in this relationship.

Certain parental characteristics, for example, education and occupation, are associated with school travel choices. For instance, higher parental educational attainment represents advantages in terms of income, with those parents potentially more likely to deliver their children to school using private vehicles (15, 18, 21). Subsequently, this could affect their children's active lifestyles. Nonetheless, whether the relationship of ATS with PA and ST varies according to parental characteristics has not yet been investigated. This is necessary to advance the knowledge in this field and capture information regarding the finer practical implications for adolescents affected by various parental characteristics. Although current findings from ATS-related studies among Chinese adolescents are inspiring, researches on the relationships of AST with PA and ST targeting Chinese samples are still scarce. Especially, little is known about how AST is associated with ST among Chinese adolescents. In addition, limited evidence is available on the relationship of ATS with PA and ST about socio-demographic factors, including individual and parental characteristics.

Therefore, as a means of contributing to resolving the evidence shortcomings in the extant literature and to develop an evidence foundation for PA- and ST-focused interventions for Chinese adolescents, this research aimed to analyse the relationship of ATS with PA and ST by the individual and parental characteristics among adolescents in China.

## Methods

### Study Design and Research Ethics

This cross-sectional study was a questionnaire survey, undertaken between May and June 2019.The sample of participants was selected in Hubei province of China. Contact was made with the commission of education in 13 cities of Hubei province. Applying a convenience sampling method, we invited three public schools (one primary school, one middle school, and one high school) per city to participate in this survey. Through the administrative support provided by the commissions of education, 3rd to 12th-grade students in 39 public primary, middle, and high schools were selected across all the cities. In total, 6,583 students (aged 10–18 years, boys: 50.9%) were invited to participate in the survey. Of these, 5,898 responses with a complete self-reported questionnaire, providing a response rate of 89.6%. The research protocol and procedure were approved by the Institutional Review Board (IRB) of the Wuhan University of Technology in March 2019. The student participants and their legal guardians provided written consent. The anonymity and confidentiality of participants were ensured following the *Declaration of Helsinki*.

### Measurements

#### Sociodemographic Information

The participants were asked, during their break time on school days, to provide self-reported data regarding sex (1 = boy, 2 = girl), grade (from 4 to 12) and current living environment (1 = urban, 2 = rural). A self-reported questionnaire was implemented to collect information from parents, including parental educational attainment (less than college/university; college/university, or higher), and occupation (office worker or manual labourer). The parents completed the paper-based questionnaire at home using a pencil.

#### Exposures (ATS)

A single-item question was included for measuring the ATS, which asked the participants: “In the last 7 days, how did you usually get to your school?” (23). This measure has been used in previously published studies (23). Participants were asked to indicate which of the travel modes represented their principal means of travelling to school and returning home. They were only given the option to select one principal transport mode, and data relating to multimode journeys (walking to or from public transport hubs, for example) was not obtained. Participants could select the following options as their responses: (1) walking independently; (2) cycling independently; (3) walking with parents or guardians; (4) cycling with parents or guardians; (5) taking school bus; (6) delivered by parents using automobile/private car/other motorised instruments; (7) other public transportation, such as public bus/underground/taxi. Overall, participants selecting options from 1 to 4 were considered *active*, whereas others were considered *passive*. According to the type of ATS, participants selecting 1 and 3 were regarded as walking; 2 and 4 were regarded as cycling; 5 were regarded as using school bus; 6 were regarded as delivered by parents, and 7 were regarded as using public transportation.

#### Outcomes (PA and ST)

PA was measured using the items derived from the Health Behaviour School-aged Children (HBSC) questionnaire, which has acceptable validity and reliability in the Chinese context (31). Two items collected the information on PA, namely, (1) “How many days did you engage in moderate to vigorous PA (MVPA) for at least 60 min on weekdays over the past week?”, with responses: 0 = none, 1 = 1 day, 2 = 2 days, 3 = 3 days, 4 = 4 days, 5 = 5 days; and (2) “How many days did you engage in MVPA for at least 60 min during the weekend over the past week?”, where responses included: 0 = none, 1 = 1 day, 2 = 2 days. Consistent with the Canadian 24-h Movement Guideline (3), sufficient PA following the definition of fulfilling the MVPA recommendation was equivalent to the participants reporting 60 min of MVPA daily for 7 days.

The following items of the HBSC questionnaire were used to obtain information relating to ST (31): (1) “How many hours did you spend watching TV or movies during your leisure time on weekdays and the weekend over the past week?” (responses: 1 = none, 2 = around half an hour, 3 = around 1 h, 4 = around 2 h, 5 = around 3 h or more); (2) “How many hours did you spend playing computer games during your leisure time on weekdays and on the weekend over the past week?” (responses: same as the above item); and (3) “How many hours did you spend on activities using electronic screen-based devices during your leisure time on weekdays and the weekend over the past week?” (responses: same as the above item). Consistent with the Canadian 24-h Movement Guidelines (10), limited ST was considered to be daily screen time of participants—including time watching TV/movies, playing computer games, and using electronic screen-based devices—of <2 h per day.

### Statistical Analysis

Before initiating the formal analysis process, all responses with missing data were omitted from the sample. We opted to concentrate our analysis on 10- to 18- year-old adolescents (grades 7–12) only, with data about further school grades being excluded. Ultimately, the final sample size, included in the analysis, was 4,128. Descriptive statistical analysis was applied to report the percentage of sociodemographic variables (e.g., sex, age, living environment, parental occupation, and education), exposures (ATS: passive vs. active; its types: school bus, delivery by parents, or public transportation as passive, or walking or cycling as active) and outcomes (PA: sufficient vs. insufficient; ST: limited vs. excessive). A Chi-square test was performed to investigate the difference in PA and ST by ATS (its types), alongside the individual as well as parental characteristics. Logistic regression was undertaken to analyse the relationship of ATS and its types (exposure) with PA and ST (outcome). All variables were incorporated into the regression analysis as categorical variables. This research presents the logistic regression results as odds ratios (ORs) with 95% confidence intervals (CIs). When examining the relationship of ATS and its types with PA and ST, all sociodemographic variables were adjusted in the models, and *p* < 0.05 was established as the level of statistical significance. The statistical analyses procedures were performed using SPSS 24.0 (IBM Corp, Chicago, IL, USA).

## Results

Table 1 presents the characteristics of participants in this study. Of all the included participants (*n* = 4,128), boys accounted for 50.9%. Younger adolescents accounted for over 60% of the participants. Over 70% of the participants lived in urban areas. Approximately 70% of participants had parents who were office worker or had an education degree of less than college/university. The proportion of participants using the passive mode of ATS was 52.7%. Regarding the types of ATS, participants who walked to school accounted for 34.1% (the largest proportion), while participants using the school bus made up the smallest proportion (0.8%). The prevalence of sufficient PA and limited ST was 17.3 and 67.9%, respectively.

**Table 1:** Characteristics of participants in this study.

	***n***	**%**
Overall	4,128	100
**Sex**		
Boy	2,101	50.9
Girl	2,027	49.1
**Age group**		
Younger adolescent	2,555	61.9
Older adolescent	1,573	38.1
**Geographical location**		
Urban	3,059	74.1
Rural	1,069	25.9
**Parental occupation**		
Officer worker	2,881	69.8
Manual labourer	1,247	30.2
**Parental education level**		
Less than college/university	2,935	71.1
College/university or higher	1,193	28.9
**Travel to school**		
Passive	2,175	52.7
Active	1,953	47.3
**Modes of travel to school**		
School bus	33	0.8
Delivered by parents	1,102	26.7
Public transportation	1,040	25.2
Walking	1,408	34.1
Cycling	545	13.2
MVPA^*^		
Insufficient	3,414	82.7
Sufficient	714	17.3
ST^**^		
Excessive	1,325	32.1
Limited	2,803	67.9

* *MVPA Moderate to vigorous physical activity, insufficient denotes not meeting the guidelines; sufficient denotes meeting the guidelines (4)*.

** *ST Screen time, excessive denotes not meeting the guidelines; limited denotes meeting the guidelines (4)*.

In Table 2, the results of the difference in PA and ST by ATS and its types are presented. Participants with active ATS showed a higher percentage of sufficient PA compared to their counterparts (20.1 vs. 14.8%, *p* < 0.001). Participants taking cycling as ATS mode had the highest percentage of sufficient PA compared to others (23.0%, *p* < 0.001). There was no significant difference in the levels of limited ST among participants with different ATS groups. Participants taking the school bus as their mode of ATS had the lowest percentage of limited ST compared to those using other types of ATS (53.3%, *p* < 0.001).

**Table 2:** Difference of PA and ST by ATS and its types.

	**Sufficient PA** **(***n*** = 714)**	***p***	**Limited ST** **(***n*** = 2,803)**	***p***
	**%**		**%**	
**ATS**				
Passive	14.8	0.000	68.4	0.213
Active	20.1		67.3	
**Types of ATS**				
School bus	15.2	0.000	53.3	0.000
Delivered by parents	15.9		73.0	
Public transportation	13.6		64.0	
Walking	19.0		69.5	
Cycling	23.0		61.7	

*ATS, Active travel to school; PA, Physical activity; ST, screen time*.

The results of the difference of PA and ST by ATS and its types as well as individual characteristics are shown in Table 3. Regardless of sex, and living environment, a difference in PA was observed between the passive and active ATS groups as well as the types of ATS (all *p* < 0.005). However, there was no significant difference in PA between the passive and active ATS groups among older adolescents (*p* = 0.116). Further, a significant difference was observed across groups of different types of ATS irrespective of sex, age, and living environment for limited ST. However, there was no significant difference of ST across groups of ATS except for younger boys (*p* < 0.001).

**Table 3:** Difference of PA and ST by ATS and its types as well as individual characteristics.

	**Sufficient PA** **(***n*** = 714)**				**Limited ST** **(***n*** = 2,803)**			
	**%**	***p***	**%**	***p***	**%**	***p***	**%**	***p***
	**Boy**		**Girl**		**Boy**		**Girl**	
**ATS**								
Passive	17.8	0.000	12.3	0.040	66.5	0.562	70.0	0.621
Active	24.6		14.1		65.7		69.4	
**Types of ATS**								
School bus	18.2	0.000	10.8	0.010	58.2	0.000	45.9	0.000
Delivered by parents	19.6		13.3		70.8		74.5	
Public transportation	16.3		10.9		63.1		64.9	
Walking	24.5		13.4		68.7		70.3	
Cycling	24.9		17.5		60.5		65.2	
	**Younger adolescents**		**Older adolescents**		**Younger adolescents**		**Older adolescents**	
**ATS**								
Passive	17.4	0.000	11.6	0.116	72.0	0.010	63.9	0.559
Active	23.2		13.2		69.2		63.1	
**Types of ATS**								
School bus	12.5	0.000	17.3	0.002	55.0	0.000	51.9	0.000
Delivered by parents	17.3		12.6		73.5		71.8	
Public transportation	17.7		10.9		69.8		60.0	
Walking	21.9		11.0		70.9		65.5	
Cycling	27.8		16.6		63.6		59.2	
	**Urban**		**Rural**		**Urban**		**Rural**	
**ATS**								
Passive	14.9	0.000	14.6	0.000	68.5	0.050	68.0	0.414
Active	19.5		21.8		66.5		69.4	
**Types of ATS**								
School bus	16.9	0.000	12.1	0.000	55.9	0.000	48.5	0.000
Delivered by parents	16.2		14.9		73.3		72.2	
Public transportation	13.4		14.3		63.9		64.2	
Walking	17.9		21.9		68.7		71.8	
Cycling	23.5		21.7		61.0		63.6	

*ATS, Active travel to school; PA, Physical activity; ST, screen time*.

In Table 4, more participants with an active ATS had sufficient PA regardless of parental education levels compared to those with a passive ATS (less than college/university: 20.7% > 14.6%, *p* < 0.001; college/university or higher: 18.7% > 15.2%, *p* = 0.006), and particularly, participants who selected cycling had the highest percentages compared with the other ATS types. In terms of limited ST, participants who were delivered by parents had the highest percentages (less than college/university: 70.6%, *p* < 0.001; college/university or higher: 77.9%, *p* = 0.004). Similar to the parental education level groups, more participants using an active form of travel to school had sufficient PA regardless of their parent's occupations compared with those with passive ATS (office worker: 20.4% > 14.9%, manual labourer: 19.6% > 14.6%, both *p* < 0.001), and participants using cycling had the highest percentages compared with other ATS types (both 23.0%, both *p* < 0.001). Participants who were delivered by parents had the highest percentages of limited ST compared to those with other ATS types (office worker: 74.6%, manual labourer: 68.6%, both *p* < 0.001).

**Table 4:** Difference of PA and ST by travel to school and its types as well as parental characteristics.

	**Sufficient PA**				**Limited ST**			
	**%**	***p***	**%**	***p***	**%**	***p***	**%**	***p***
**Parental education**	**Less than college/university**		**College/university or higher**		**Less than college/university**		**College/university or higher**	
**ATS**								
Passive	14.6	0.000	15.2	0.006	65.5	0.819	75.4	0.076
Active	20.7		18.7		65.2		72.7	
**Types of ATS**								
School bus	13.6	0.000	18.2	0.005	45.8	0.000	66.7	0.004
Delivered by parents	15.8		16.0		70.6		77.9	
Public transportation	13.5		14.0		61.1		72.3	
Walking	19.7		17.3		67.7		74.1	
Cycling	23.3		22.3		58.7		69.1	
**Parental occupation**	**Officer worker**		**Manual labourer**		**Officer worker**		**Manual labourer**	
**ATS**								
Passive	14.9	0.000	14.6	0.000	70.3	0.833	63.7	0.205
Active	20.4		19.6		70.1		61.6	
**Types of ATS**								
School bus	16.9	0.000	11.1	0.000	60.0	0.000	37.0	0.000
Delivered by parents	15.2		17.7		74.6		68.6	
Public transportation	14.4		11.8		65.8		59.9	
Walking	19.3		18.4		71.8		64.8	
Cycling	23.0		23.0		65.7		52.8	

*ATS, Active travel to school; PA, Physical activity; ST, screen time*.

In Table 5, the results from the logistic regression model revealing the relationships of ATS with PA and ST are shown. Overall, participants with ATS were more likely to have sufficient PA (OR = 1.26, 95% CI: 1.14–1.39). In examining the relationships of ATS with PA and ST by sex, only boys with ATS had a higher chance of being sufficiently active (OR = 1.41, 95% CI: 1.24–1.61), compared with their counterparts. Significant relationships were also observed in participates of the younger age group (OR = 1.35, 95% CI: 1.20–1.52) and those whose parental education level was less than college/university (OR = 1.30, 95% CI: 1.16–1.47). Regardless of living environment and parental occupation, participants with ATS were more likely to have sufficient PA (all OR > 1.20). However, participants with ATS showed a non-significant relationship with ST.

**Table 5:** The relationships of travel (modes) to school with MVPA and ST by different characteristics.

	**MVPA**						**ST**					
	**OR**	**95% CI**		**OR**	**95% CI**		**OR**	**95% CI**		**OR**	**95% CI**	
Active	**1.26**	**1.14**	**1.39**				0.94	0.86	1.01			
Passive	Ref						Ref					
	**Boys**			**Girls**			**Boys**			**Girls**		
Active	**1.41**	**1.24**	**1.61**	1.05	0.90	1.23	0.93	0.83	1.03	0.94	0.84	1.06
Passive	Ref			Ref			Ref			Ref		
	**Younger adolescents**			**Older adolescents**			**Younger adolescents**			**Older adolescents**		
Active	**1.35**	**1.20**	**1.52**	1.07	0.88	1.29	0.91	0.82	1.01	0.98	0.86	1.12
Passive	Ref			Ref			Ref			Ref		
	**Urban**			**Rural**			**Urban**			**Rural**		
Active	**1.23**	**1.09**	**1.38**	**1.40**	**1.15**	**1.70**	0.89	0.81	0.98	0.91	1.07	1.25
Passive	Ref			Ref			Ref			Ref		
	**Less than college/university**			**College/university or higher**			**Less than college/university**			**College/university or higher**		
Active	**1.30**	**1.16**	**1.47**	1.16	0.96	1.39	0.98	0.89	1.07	0.84	0.72	0.99
Passive	Ref			Ref			Ref			Ref		
	**Officer worker**			**Manual labourer**			**Officer worker**			**Manual labourer**		
Active	**1.26**	**1.11**	**1.41**	**1.26**	**1.05**	**1.51**	0.95	0.86	1.05	0.90	0.78	1.04
Passive	Ref			Ref			Ref			Ref		

*MVPA, Moderate to vigorous physical activity; ST, Screen time*.

*Bold fonts denote statistical significance*.

Table 6 shows the results from the regression model for the relationships of types of ATS with PA and ST by individual and parental characteristics. In general, participants who selected walking or cycling were more likely to have sufficient PA overall, in boy, younger adolescents, and urban participants, and those who had parents with less than college/university education level or office worker occupation. Conversely, cycling had a likelihood of increasing the sufficient PA among girls, older adolescents, or those who had parents with a higher education level (college/university or higher) or manual labourer. Concerning the relationship of types of ATS with ST, participants with walking (OR = 1.17, 95% CI: 1.05–1.30) and delivered by parents (OR = 1.34, 95% CI: 1.19–1.50) were more likely to have limited ST. Significant relationships were also found among boys and girls, older adolescents, those living in rural areas, those having parents with less than college/university education level, or with office workers.

**Table 6:** The relationships of types of active travel to school with MVPA and ST by different characteristics.

	**MVPA**						**ST**					
	**OR**	**95% CI**		**OR**	**95% CI**		**OR**	**95% CI**		**OR**	**95% CI**	
Walking	**1.23**	**1.07**	**1.41**				**1.17**	**1.05**	**1.30**			
Cycling	**1.52**	**1.29**	**1.79**				**0.88**	**0.77**	**0.99**			
School bus	1.06	0.59	1.89				**0.60**	**0.39**	**0.92**			
Delivered by parents	1.06	0.92	1.23				**1.34**	**1.19**	**1.50**			
Public transportation	Ref						Ref					
	**Boys**			**Girls**			**Boys**			**Girls**		
Walking	**1.41**	**1.18**	**1.69**	0.99	0.79	1.23	**1.16**	**1.01**	**1.34**	**1.17**	**1.01**	**1.37**
Cycling	**1.57**	**1.29**	**1.91**	**1.47**	**1.07**	**2.00**	**0.83**	**0.70**	**0.98**	0.97	0.77	1.23
School bus	1.10	0.54	2.22	0.98	0.34	2.82	0.75	0.43	1.30	**0.43**	**0.22**	**0.83**
Delivered by parents	1.07	0.88	1.32	1.01	0.81	1.27	**1.25**	**1.06**	**1.48**	**1.42**	**1.21**	**1.66**
Public transportation	Ref			Ref			Ref			Ref		
	**Younger adolescents**			**Older adolescents**			**Younger adolescents**			**Older adolescents**		
Walking	**1.29**	**1.08**	**1.53**	1.04	0.81	1.33	1.06	0.91	1.23	**1.23**	**1.04**	**1.44**
Cycling	**1.56**	**1.26**	**1.93**	**1.34**	**1.03**	**1.74**	**0.76**	**0.63**	**0.92**	0.99	0.82	1.20
School bus	0.65	0.25	1.69	1.71	0.81	3.60	**0.47**	**0.25**	**0.90**	0.69	0.39	1.20
Delivered by parents	1.01	0.84	1.22	1.23	0.95	1.58	1.15	0.98	1.34	**1.62**	**1.36**	**1.92**
Public transportation	Ref			Ref			Ref			Ref		
	**Urban**			**Rural**			**Urban**			**Rural**		
Walking	**1.21**	**1.03**	**1.42**	1.25	0.95	1.64	1.11	0.98	1.26	**1.33**	**1.08**	**1.65**
Cycling	**1.66**	**1.38**	**2.01**	1.09	0.78	1.51	0.86	0.74	1.01	0.94	0.73	1.23
School bus	1.17	0.58	2.35	0.95	0.32	2.83	0.65	0.38	1.09	0.52	0.26	1.05
Delivered by parents	1.17	0.98	1.38	0.77	0.56	1.04	**1.36**	**1.19**	**1.55**	**1.30**	**1.03**	**1.65**
Public transportation	Ref			Ref			Ref			Ref		
	**Less than college/university**			**College/university or higher**			**Less than college/university**			**College/university or higher**		
Walking	**1.22**	**1.04**	**1.43**	1.22	0.93	1.60	**1.25**	**1.11**	**1.42**	0.97	0.78	1.20
Cycling	**1.50**	**1.24**	**1.82**	**1.53**	**1.12**	**2.09**	0.90	0.77	1.05	0.82	0.63	1.07
School bus	0.97	0.45	2.09	1.29	0.52	3.24	**0.53**	**0.32**	**0.90**	0.70	0.33	1.48
Delivered by parents	0.99	0.83	1.18	1.23	0.93	1.62	**1.40**	**1.23**	**1.60**	1.19	0.95	1.49
Public transportation	Ref			Ref			Ref			Ref		
	**Officer worker**			**Manual labourer**			**Officer worker**			**Manual labourer**		
Walking	**1.19**	**1.01**	**1.41**	**1.30**	**1.01**	**1.69**	**1.17**	**1.02**	**1.33**	1.18	0.98	1.42
Cycling	**1.39**	**1.14**	**1.69**	**1.85**	**1.36**	**2.50**	0.95	0.80	1.11	**0.75**	**0.59**	**0.94**
School bus	1.09	0.56	2.12	0.92	0.27	3.15	0.73	0.44	1.22	**0.37**	**0.17**	**0.82**
Delivered by parents	0.99	0.83	1.18	1.27	0.96	1.69	**1.33**	**1.16**	**1.53**	**1.38**	**1.12**	**1.70**
Public transportation	Ref			Ref			Ref			Ref		

*MVPA, Moderate to vigorous physical activity; ST, Screen time*.

*Bold fonts denote statistical significance*.

## Discussion

To the best of our knowledge, this study is one of the first cross-sectional investigations into the relationship of ATS and its types with PA and ST among adolescents of the Chinese samples. This research analyses the correlation of ATS and its types with PA and ST about different individual and parental characteristics, which potentially offers significant practical implications and advances the knowledge in this field.

### Summary of Findings

In this current study, the proportion of the sample engaging in ATS was 47.3%, with participants who walked (34.1%) constituting a greater percentage than those who cycled (13.2%). Regarding the question relating to levels of adequate PA and limited ST among the adolescents, only 17.3% attained the former, whereas the latter was under acceptable levels. Furthermore, we found that participants engaged in ATS had a greater chance of also participating inadequate PA levels, while ATS was not connected with limited ST. Regarding types of ATS, participants engaged in walking or cycling both had a higher chance of undertaking sufficient PA rather than being characterised by limited ST. The relationships of ATS and its types with PA and ST differed according to individual and parental characteristics.

### Interpretations of Findings

Low PA levels among adolescents have been demonstrated across the literature (2, 4, 32). The present research indicated that only ~20% of adolescents participated in the recommended level of PA [60 min of moderate to vigorous PA daily (5)], indicating a lower level of PA among adolescents. This result is consistent with the previously published studies of a regional nature (33, 34). Nevertheless, in contrast with the nationally representative surveys in China (32), the PA level established in the present study is lower. This reduced level is potentially explained by the varying measures adopted, which possibly produced inconsistencies in terms of estimating the amount of PA. Concerning the level of ST among adolescents in this study, our level is following studies based on Chinese adolescent samples (32–34). Despite the ST level exceeding 60%, limiting ST is a necessary mission for the promotion of adolescent health. Likewise, given the health benefits linked to sufficient PA among adolescents (7, 10), increased PA is further imperative. The Social-Ecological Model has summarised the correlates of PA and ST (29). Nevertheless, given the significant variations in China's economy, society and geography, investigating the correlates and determinants of PA and ST among adolescents across the country's various regions requires additional research (33).

#### Levels in Active Travel to School

The current study indicates that just below half of the participants (47.3%) engaged in ATS from home to school, which corroborates other studies on Chinese (23, 24) and North American samples (35, 36). Regardless, in contrast with studies of European countries (37, 38), the adolescents in our study showed reduced ATS performance. It is probable that the difference between our study and other research in ATS levels due to various measures used. Nevertheless, the ATS among Chinese adolescents has declined over the past decade (24), suggesting that they are less physically active (through walking or cycling). From this perspective, the ATS level among adolescents in our study is potentially unsatisfactory. This is a somewhat concerning situation that should be explained about local contexts and across various regions or cities (39). Given the health benefits of ATS for adolescents (30), the promotion of ATS among adolescents is to be encouraged.

Our research is one of the few investigations assessing types of ATS among Chinese adolescents. Among the participants engaged in ATS, the majority walked between school and home. This finding is consistent with previous studies (23). A Study in 2010 by Sun et al. found that 40.1 and 41.4% of Chinese 7–9 grader and 10–12 graders walked to school, while only 14.5 and 9.5% of 7–9 grader and 10–12 graders cycled to school. Our results showed lower percentages of walking as well as cycling. This declining trend had been indicated by prior research. For instance, Yang et al. (24) clarified that participation in walking or cycling between home and school has declined over the years. Presently, there is a dearth of understanding regarding walking or cycling trends among Chinese adolescents at the national or regional level. We suggest that future studies should seek to more effectively explore the patterns of either walking or cycling, thus providing evidence-based information for promoting ATS.

#### Overall Associations of ATS With PA and ST

Reflecting the findings of numerous previous studies (16, 21, 27, 28), the current study reaffirms that participants engaged in ATS have a greater chance of participating insufficient PA. Potential reasons may be that (1) ATS is an aspect of daily PA; thus, participants engaged in ATS are more likely to report higher PA; (2) participants engaged in ATS were similarly or more intensely engaged in other types of PA. Collectively, these two potential reasons may be responsible for the association of ATS with sufficient PA. Nevertheless, the ATS and PA were affected by different variables across various regions.

Accordingly, the mechanism connecting ATS and PA varies depending upon the social and environmental factors. Therefore, it is advocated that further studies should investigate the correlation of ATS and PA to a greater extent, implementing enhanced study designs and multiple data sources. Nevertheless, this study finds that ATS was not associated with ST among adolescents. A systematic review indicated that the relationship of ATS with ST was inconsistent across the literature (14), meaning the correlation between these two variables remains ambiguous. However, informed by the findings of this study, ATS potentially plays a role in promoting PA, as opposed to diminishing ST among adolescents.

#### Variations in the Association Between ATS and PA

We further discovered that the relationship of ATS with PA varies according to sex, age, and parental education. Specifically, the relationship between ATS and PA was found to be significant among boys, suggesting that only boys engaging in ATS have potentially involved insufficient PA (15). However, such a significant relationship was not observed among girls. This may be potential because parents grant greater freedom to boys to engage in ATS as opposed to girls. Thus, girls may be exposed to fewer opportunities to engage in ATS behaviour, ultimately restricting their ability to engage in sufficient PA levels. Moreover, the sex difference in the relationship of ATS with PA was similar to the age difference.

The present research found that younger adolescents, as opposed to their older peers were engaged more in ATS r and had a greater chance of also participating insufficient PA. This result is explainable according to the Chinese context. In our study, older adolescents were those in grades 10–12, which is a vital period that covers the college entrance test. During this period, adolescents tend to be delivered to school by their parents for time-saving reasons, allowing a greater amount of time to be spent on studying. Conversely, such a situation would not arise among younger adolescents due to lower academic pressure.

Among participants whose parents had lower educational attainment, those engaged in ATS had a greater prospect of attaining sufficient PA levels. Nevertheless, this significant relationship was not detected among participants whose parents had higher educational attainment levels. A potential reason is that parents with lower levels of educational attainment have lower levels of income compared with their counterparts. Consequently, it is less likely that those parents possess automobiles, which potentially causes their children to travel between home and school through walking or cycling.

This research has established that the relationships between types of ATS with PA vary according to individual and parental characteristics. We primarily identified that participants engaged in walking or cycling had a greater likelihood of participating insufficient PA compared with their counterparts. Unsurprisingly, when assessing the odds ratio of walking and cycling about sufficient PA, the former is lower than the latter. This finding is consistent with the results of the study of Roth et al. (15), where the association between cycling and overall PA (OR = 1.93) was stronger than that between walking and overall PA (OR = 1.17). Another study also provided a similar finding that there was a difference in the PA level between active walkers and cyclists (40). However, there is still no well-recognised explanation for this finding and more research is required into the social and environmental determinants of ATS with PA.

A similar relationship was identified among boys, younger adolescents, participants whose parents had reduced education levels or were office workers. Significantly, only girls engaged in cycling showed sufficient PA in our study, providing an inconsistency with the results for boys. Future studies should attempt to answer this difference for the relationship of types of ATS with PA based on the different subpopulations' characteristics and other factors.

A noticeable distinction is apparent in the relationship of types of ATS with PA according to the living environment. Specifically, in urban areas, adolescents engaged in cycling were more likely to be involved insufficient PA, whereas adolescents in rural areas who were engaged in walking had a greater prospect of undertaking sufficient PA. Unfortunately, no data provided by our study were able to clarify the variation. The differences in built environment between urban and rural settings may provide a plausible explanation for this. A similar finding was established for participants with different parental education levels and occupations. Given the limited evidence across the extant literature, more studies should address these research questions in the future.

#### Variations in the Association Between ATS and ST

When looking at the difference of individual and parental characteristics in the relationship of types of ATS with limited ST, some novel findings should be mentioned. In the current study, participants who walked and were delivered by parents had higher odds of having limited ST compared with their counterparts. To our knowledge, no data provided by previous studies were comparable with our study findings. Some possible reasons for explaining the findings are that (1) participants who walked to school could be regarded as a lower level of socio-economic status, and may not be able to afford as many screen-based devices, ultimately reducing their ST; (2) participants delivered by parents would be exposed to much stricter supervision that limits the time spent for screen-based activities. Owing to less research, more studies should confirm our assumptions. In addition to the overall findings, the individual and parental differences in the relationship of types of ATS with ST were found. Such variations should be explained by more factors, whereas the present study failed to provide this indicative information, future studies should answer the variations found by our study, which can provide more specific practical implications.

### Implications and Future Recommendations for Research

Irrespective of the preliminary nature of the evidence regarding the variations in the relationship of ATS and its types with individual and parental characteristics, the present study affirmed the role of ATS in promoting PA among adolescents. This research has expanded on prior research, demonstrating that individual and parental characteristics show different relationships regarding ATS compared to PA. Nevertheless, given that ATS and PA are two complex behaviours that are affected by numerous variables, the relationships of ATS and its types with PA according to various characteristics requires further replication and clarification of the results to provide more robust evidence. Practically, the current study should prove beneficial in encouraging adolescent PA through ATS, with the design of effective policies and actions being necessary.

When designing ATS interventions for enhancing PA among adolescents, individual and parental characteristics must be considered. It is recommended that future research should concentrate on the mechanisms linking ATS with PA within various contexts (for example sex and living environment). Based on the cross-sectional nature of our research, prospective longitudinal studies are necessary to confirm the relationships we observed, as well as to elucidate whether a potential causal relationship is apparent between ATS and PA. Moreover, it is necessary to undertake further experimental research to analyse the extent to which ATS interventions offer further effectiveness for promoting PA among adolescents.

### Study Strengths and Limitations

This study offered certain advantages. First, the study adopted a relatively large sample size as a means of investigating the relationship of ATS with PA and ST, thus enhancing the generalizability of research findings. Second, the present study is one of the very limited number of investigations analysing the associations of ATS with PA and ST as they relate to different individual and parental characteristics. The research findings are potentially beneficial for designing specific PA and ST interventions. However, there are certain limitations to this study that should be clarified. Due to the cross-sectional nature of the study, the findings of the study should be interpreted with caution. Moreover, our study used a self-reported questionnaire to assess the ATS, PA, and ST, which was subjective to recall bias of measurement. Third, this study did not include/explore more potential confounders, such as time spent during ATS and car ownership, that may affect the relationship of ATS with PA and ST. Fourth, to better explain the relationship of ATS with PA or ST, additional psychological (e.g., attitude towards ATS), social (e.g., safety and parental awareness of ATS) and physical environmental (e.g., distance to school) factors should be considered for more reliable interpretations. Finally, the ATS and PA, as well as ST, are also affected by other factors, such as income level. We recommend further study of the relationship of ATS with PA and ST by income and other sociodemographic factors, particularly of longitudinal nature. Future studies should address these limitations to provide an improved evidence base.

## Conclusions

Overall, our study indicated that approximately half of the adolescents engaged in ATS, with a majority of participants preferring to travel through cycling between home and school. ATS among adolescents was linked with sufficient PA as opposed to ST. Therefore, the relationships of ATS and its types with PA require further clarification by different contexts, including sex, age differences, and parental characteristics. Nevertheless, this study retains its specific significance to the implementation of PA promotional activities among adolescents.

## Data Availability Statement

The raw data supporting the conclusions of this article will be made available by the authors, without undue reservation.

## Ethics Statement

The studies involving human participants were reviewed and approved by Wuhan University of Technology. Written informed consent to participate in this study was provided by the participants' legal guardian/next of kin.

## Author Contributions

CH, YL, and S-TC conceptualised and designed this study. CH, JY, and YL analysed interpreted data and drafted the manuscript. AM and S-TC provided important intellectual roles in revision. All authors read and approved the final manuscript.

## Conflict of Interest

The authors declare that the research was conducted in the absence of any commercial or financial relationships that could be construed as a potential conflict of interest.

## Publisher's Note

All claims expressed in this article are solely those of the authors and do not necessarily represent those of their affiliated organizations, or those of the publisher, the editors and the reviewers. Any product that may be evaluated in this article, or claim that may be made by its manufacturer, is not guaranteed or endorsed by the publisher.

## References

[1] Shen H Yan J Hong J-T Clark C Yang X-N Liu Y. Prevalence of physical activity and sedentary behavior among Chinese children and adolescents: variations, gaps, and recommendations. Int J Environ Res Public Health. (2020) 17:3066. 10.3390/ijerph17093066 32354193 PMC7246713

[2] Hallal PC Andersen LB Bull FC Guthold R Haskell W Ekelund U. Global physical activity levels: surveillance progress, pitfalls, and prospects. Lancet. (2012) 380:247–57. 10.1016/S0140-6736(12)60646-1 22818937

[3] Tremblay MS Barnes JD González SA Katzmarzyk PT Onywera VO Reilly JJ. Global matrix 2.0: report card grades on the physical activity of children and youth comparing 38 countries. J Phys Activity Health. (2016) 13:S343–66. 10.1123/jpah.2016-0594 27848745

[4] Guthold R Stevens GA Riley LM Bull FC. Global trends in insufficient physical activity among adolescents: a pooled analysis of 298 population-based surveys with 1 6 million participants. Lancet Child Adolesc Health. (2020) 4:23–35. 10.1016/S2352-4642(19)30323-2 31761562 PMC6919336

[5] Tremblay MS Carson V Chaput J-P Connor Gorber S Dinh T Duggan M. Canadian 24-hour movement guidelines for children and youth: an integration of physical activity, sedentary behaviour, and sleep. Appl Physiol Nutr Metab. (2016) 41:S311–27. 10.1139/apnm-2016-0151 27306437

[6] Chen S-T Yan J. Prevalence and selected sociodemographic of movement behaviors in schoolchildren from low-and middle-income families in Nanjing, China: a cross-sectional questionnaire survey. Children. (2020) 7:13. 10.3390/children7020013 32069924 PMC7072474

[7] Janssen LeBlanc AG. Systematic review of the health benefits of physical activity and fitness in school-aged children and youth. Int J Behav Nutr Phys Activity. (2010) 7:1–16. 10.1186/1479-5868-7-40 20459784 PMC2885312

[8] Tremblay MS LeBlanc AG Kho ME Saunders TJ Larouche R Colley RC. Systematic review of sedentary behaviour and health indicators in school-aged children and youth. Int J Behav Nutr Phys Activity. (2011) 8:1–22. 10.1186/1479-5868-8-98 21936895 PMC3186735

[9] Carson V Hunter S Kuzik N Gray CE Poitras VJ Chaput J-P. Systematic review of sedentary behaviour and health indicators in school-aged children and youth: an update. Appl Physiol Nutr Metab. (2016) 41:S240–65. 10.1139/apnm-2015-0630 27306432

[10] Poitras VJ Gray CE Borghese MM Carson V Chaput J-P Janssen I. Systematic review of the relationships between objectively measured physical activity and health indicators in school-aged children and youth. Appl Physiol Nutr Metab. (2016) 41:S197–239. 10.1139/apnm-2015-0663 27306431

[11] Chaput J Leduc G Boyer C Belanger P LeBlanc A Borghese M. Objectively measured physical activity, sedentary time and sleep duration: independent and combined associations with adiposity in canadian children. Nutr Diabetes. (2014) 4:e117. 10.1038/nutd.2014.14 24911633 PMC4079924

[12] Biddle SJ O'Connell S Braithwaite RE. Sedentary behaviour interventions in young people: a meta-analysis. Br J Sports Med. (2011) 45:937–42. 10.1136/bjsports-2011-090205 21807671

[13] Heath GW Parra DC Sarmiento OL Andersen LB Owen N Goenka S. Evidence-based intervention in physical activity: lessons from around the world. Lancet. (2012) 380:272–81. 10.1016/S0140-6736(12)60816-2 22818939 PMC4978123

[14] Schoeppe S Duncan MJ Badland H Oliver M Curtis C. Associations of children's independent mobility and active travel with physical activity, sedentary behaviour and weight status: a systematic review. J Sci Med Sport. (2013) 16:312–9. 10.1016/j.jsams.2012.11.001 23219100

[15] Roth MA Millett CJ Mindell JS. The contribution of active travel (walking and cycling) in children to overall physical activity levels: a national cross sectional study. Prevent Med. (2012) 54:134–9. 10.1016/j.ypmed.2011.12.004 22182478

[16] Barranco-Ruiz Y Guevara-Paz AX Ramírez-Vélez R Chillón P Villa-González E. Mode of commuting to school and its association with physical activity and sedentary habits in young Ecuadorian students. Int J Environ Res Public Health. (2018) 15:2704. 10.3390/ijerph15122704 30513629 PMC6313456

[17] Sahlqvist S Song Y Ogilvie D. Is active travel associated with greater physical activity? The contribution of commuting and non-commuting active travel to total physical activity in adults. Prevent Med. (2012) 55:206–11. 10.1016/j.ypmed.2012.06.028 22796629 PMC3824070

[18] Nilsson Andersen LB Ommundsen Y Froberg K Sardinha LB Piehl-Aulin K Ekelund U. Correlates of objectively assessed physical activity and sedentary time in children: a cross-sectional study (The European Youth Heart Study). BMC Public Health. (2009) 9:1–7. 10.1186/1471-2458-9-322 19735565 PMC2745386

[19] Heelan KA Donnelly JE Jacobsen DJ Mayo MS Washburn R Greene L. Active commuting to and from school and BMI in elementary school children–preliminary data. Child Care Health Dev. (2005) 31:341–9. 10.1111/j.1365-2214.2005.00513.x 15840154

[20] King AC Parkinson KN Adamson AJ Murray L Besson H Reilly JJ. Correlates of objectively measured physical activity and sedentary behaviour in English children. Eur J Public Health. (2011) 21:424–31. 10.1093/eurpub/ckq104 20650946

[21] Owen CG Nightingale CM Rudnicka AR Van Sluijs EM Ekelund U Cook DG. Travel to school and physical activity levels in 9–10 year-old UK children of different ethnic origin; child heart and health study in England (CHASE). PLoS ONE. (2012) 7:e30932. 10.1371/journal.pone.0030932 22319596 PMC3272007

[22] Landsberg B Plachta-Danielzik S Much D Johannsen M Lange D Müller MJ. Associations between active commuting to school, fat mass and lifestyle factors in adolescents: the Kiel obesity prevention study (KOPS). Eur J Clin Nutr. (2008) 62:739–47. 10.1038/sj.ejcn.1602781 17522617

[23] Sun Y Liu Y Tao F-B. Associations between active commuting to school, body fat, and mental well-being: population-based, cross-sectional study in China. J Adoles Health. (2015) 57:679–85. 10.1016/j.jadohealth.2015.09.002 26592335

[24] Yang Y Hong X Gurney JG Wang Y. Active travel to and from school among school-age children during 1997–2011 and associated factors in China. J Phys Activity Health. (2017) 14:684–91. 10.1123/jpah.2016-0590 28513321

[25] Zacharias J Zhen B Han X Huang Y. Local environment and social factors in primary school children's afterschool commute in China. Preventive medicine reports. (2017) 7:206–10. 10.1016/j.pmedr.2017.06.012 28752024 PMC5524311

[26] Zacharias J Han X. Longer afterschool active commutes and the travel environment of middle schools in Shenzhen, China. Prevent Med Rep. (2018) 12:170–5. 10.1016/j.pmedr.2018.09.009 30306013 PMC6172362

[27] Gao Y Wang J-j Lau PW Ransdell L. Pedometer-determined physical activity patterns in a segmented school day among Hong Kong primary school children. J Exer Sci Fitness. (2015) 13:42–8. 10.1016/j.jesf.2015.03.002 29541098 PMC5812870

[28] Huang WY Wong SH He G. Is a change to active travel to school an important source of physical activity for Chinese children? Pediatr Exerc Sci. (2017) 29:161–8. 10.1123/pes.2016-0001 28271798

[29] Bauman AE Reis RS Sallis JF Wells JC Loos RJ Martin BW. Correlates of physical activity: why are some people physically active and others not? Lancet. (2012) 380:258–71. 10.1016/S0140-6736(12)60735-1 22818938

[30] Lubans DR Boreham CA Kelly P Foster CE. The relationship between active travel to school and health-related fitness in children and adolescents: a systematic review. Int J Behav Nutr Phys Activity. (2011) 8:1–12. 10.1186/1479-5868-8-1 21269514 PMC3039551

[31] Liu Y Wang M Tynjälä J Lv Y Villberg J Zhang Z. Test-retest reliability of selected items of health behaviour in school-aged children (HBSC) survey questionnaire in Beijing, China. BMC Med Res Methodol. (2010) 10:1–9. 10.1186/1471-2288-10-1 20696078 PMC2927607

[32] Zhu Z Tang Y Zhuang J Liu Y Wu X Cai Y. Physical activity, screen viewing time, and overweight/obesity among Chinese children and adolescents: an update from the 2017 physical activity and fitness in China—the youth study. BMC Public Health. (2019) 19:1–8. 10.1186/s12889-019-6515-9 30767780 PMC6376726

[33] Chen S-T Liu Y Hong J-T Tang Y Cao Z-B Zhuang J. Co-existence of physical activity and sedentary behavior among children and adolescents in Shanghai, China: do gender and age matter? BMC Public Health. (2018) 18:1–9. 10.1186/s12889-017-4524-0 30466431 PMC6251113

[34] Liu Y Tang Y Cao Z-B Chen P-J Zhang J-L Zhu Z. Results from Shanghai's (China) 2016 report card on physical activity for children and youth. J Phys Activity Health. (2016) 13:S124–8. 10.1123/jpah.2016-0362 27848739

[35] Bookwala Elton-Marshall T Leatherdale ST. Factors associated with active commuting among a nationally representative sample of Canadian youth. Can J Public Health. (2014) 105:e348–53. 10.17269/cjph.105.4139 25365269 PMC6972119

[36] Aubert S Barnes JD Abdeta C Abi Nader P Adeniyi AF Aguilar-Farias N. Global matrix 3.0 physical activity report card grades for children and youth: results and analysis from 49 countries. J Phys Activity Health. (2018) 15:S251–73. 10.1123/jpah.2018-0472 30475137

[37] Pizarro AN Schipperijn J Andersen HB Ribeiro JC Mota J Santos MP. Active commuting to school in Portuguese adolescents: using PALMS to detect trips. J Trans Health. (2016) 3:297–304. 10.1016/j.jth.2016.02.004

[38] Dessing D De Vries SI Graham JM Pierik FH. Active transport between home and school assessed with GPS: a cross-sectional study among Dutch elementary school children. BMC Public Health. (2014) 14:1–8. 10.1186/1471-2458-14-227 24597513 PMC3973871

[39] Panter JR Jones AP Van Sluijs EM. Environmental determinants of active travel in youth: a review and framework for future research. Int J Behav Nutr Phys Activity. (2008) 5:1–14. 10.1186/1479-5868-5-34 18573196 PMC2483993

[40] Cooper AR Andersen LB Wedderkopp N Page AS Froberg K. Physical activity levels of children who walk, cycle, or are driven to school. Am J Prev Med. (2005) 29:179–84. 10.1016/j.amepre.2005.05.009 16168866

